# Comprehensive analysis based on DNA methylation and RNA-seq reveals hypermethylation of the up-regulated WT1 gene with potential mechanisms in PAM50 subtypes of breast cancer

**DOI:** 10.7717/peerj.11377

**Published:** 2021-05-04

**Authors:** Chongyang Ren, Xiaojiang Tang, Haitao Lan

**Affiliations:** 1 Department of Breast Cancer, Guangdong Provincial People’s Hospital & Guangdong Academy of Medical Sciences, Guangzhou, Guangdong, China; 2 Department of Breast Surgery, The First Affiliated Hospital of Xi’an Jiaotong University, Xi’an, Shanxi, China; 3 Academy of Medical Sciences, Sichuan Provincial People’s Hospital, Chengdu, Sichuan, China

**Keywords:** Breast cancer, Methylation, WT1, Potential therapy target

## Abstract

**Background:**

Breast cancer (BC), one of the most widespread cancers worldwide, caused the deaths of more than 600,000 women in 2018, accounting for about 15% of all cancer-associated deaths in women that year. In this study, we aimed to discover potential prognostic biomarkers and explore their molecular mechanisms in different BC subtypes using DNA methylation and RNA-seq.

**Methods:**

We downloaded the DNA methylation datasets and the RNA expression profiles of primary tissues of the four BC molecular subtypes (luminal A, luminal B, basal-like, and HER2-enriched), as well as the survival information from The Cancer Genome Atlas (TCGA). The highly expressed and hypermethylated genes across all the four subtypes were screened. We examined the methylation sites and the downstream co-expressed genes of the selected genes and validated their prognostic value using a different dataset (GSE20685). For selected transcription factors, the downstream genes were predicted based on the Gene Transcription Regulation Database (GTRD). The tumor microenvironment was also evaluated based on the TCGA dataset.

**Results:**

We found that Wilms tumor gene 1 (*WT1*), a transcription factor, was highly expressed and hypermethylated in all the four BC subtypes. All the *WT1* methylation sites exhibited hypermethylation. The methylation levels of the TSS200 and 1stExon regions were negatively correlated with *WT1* expression in two BC subtypes, while that of the gene body region was positively associated with *WT1* expression in three BC subtypes. Patients with low *WT1* expression had better overall survival (OS). Five genes including *COL11A1*, *GFAP*,* FGF5*,* CD300LG*, and* IGFL2* were predicted as the downstream genes of *WT1*. Those five genes were dysregulated in the four BC subtypes. Patients with a favorable 6-gene signature (low expression of *WT1* and its five predicted downstream genes) exhibited better OS than that with an unfavorable 6-gene signature. We also found a correlation between *WT1* and tamoxifen using STITCH. Higher infiltration rates of CD8 T cells, plasma cells, and monocytes were found in the lower quartile *WT1* group and the favorable 6-gene signature group. In conclusion, we demonstrated that *WT1* is hypermethylated and up-regulated in the four BC molecular subtypes and a 6-gene signature may predict BC prognosis.

## Introduction

According to the World Health Organization’s report, there were approximately 2.09 million breast cancer (BC) cases in 2018, making it the second most common cancer worldwide. Almost 627,000 women died from BC in 2018, accounting for almost 15% of all cancer-associated deaths in women (https://www.who.int/news-room/fact-sheets/detail/cancer). Therefore, BC is a severe medical burden that deserves extensive study. Our study aimed to discover potential prognostic biomarkers in different types of BC and explore their potential mechanisms in an epigenetic perspective.

The recent developments in sequencing technology has raised the interests in studying of the regulatory mechanism of BC progression. While normal gene expression is regulated by an intricate genetic and epigenetic regulatory system (Arechederra et al., 2018), dysregulation of oncogenes and tumor suppressor genes occurs in tumor cells (Dawson & Kouzarides, 2012). DNA methylation plays an important role in gene expression through various epigenetic mechanisms (Karemaker & Vermeulen, 2018). In tumor tissue, DNA hypomethylation shows a disperse distribution, whereas DNA hypermethylation is concentrated on CpG-rich regions, called CpG islands (Feinberg & Tycko, 2004). Gene promoter hypermethylation results in gene repression (Xu et al., 2018), but hypermethylation in the gene body (including exons and introns) (Han et al., 2017) elevates gene expression (Jones, 2012; Renner et al., 2013; Wagner et al., 2014; Wang et al., 2016). Wang et al. found that under the action of *DNMT3B*, the fully methylated body region turned refractory to *SP1* binding, releasing *SP1* for promoter binding and driving of gene expression (Wang et al., 2016). Gene methylation in the gene body might serve as a therapeutic target of cancers (Yang et al., 2014). For example, DNA methyltransferase inhibitors, approved for older acute myeloid leukemia (AML) patients, combined with 5-aza-2’-deoxycytidine predominant synergistic gene down-regulation is associated with gene body demethylation in AML cell line (Blagitko-Dorfs et al., 2019). Although the application of DNA methylation has also shown broad therapeutic potential in BC (Downs et al., 2019; Li et al., 2019; Prajzendanc et al., 2020; Sasidharan Nair et al., 2018), fewer than twenty papers about gene body methylation in BC were found in PubMed (Croes et al., 2018; De Almeida et al., 2019; Flanagan et al., 2009; Jin et al., 2019; Kim et al., 2018; Leadem et al., 2018; Li et al., 2017b; Liu et al., 2018; Peiffer et al., 2019; Rodger et al., 2019; Shenker et al., 2015; Song et al., 2015; Stefansson et al., 2015; Taslim et al., 2012; Windhorst, Song & Gazdar, 2017). As mentioned above, gene body hypermethylation elevates gene expression (Jones, 2012; Renner et al., 2013; Wagner et al., 2014; Wang et al., 2016), so we focused on the hypermethylated and up-regulated genes in our study and investigated whether gene body methylation is correlated to gene expression.

With the hope of achieving precision medicine, researchers have applied molecular biotechnology (defined by mRNA expression of 50 genes (PAM50) (Pu et al., 2020)) to classify BC into four molecular subtypes: luminal A, luminal B, basal-like (triple-negative), and HER2-enriched. However, most of the studies about gene body methylation were performed in only one or two selected subtypes of BC (Leadem et al., 2018; Liu et al., 2018; Peiffer et al., 2019; Rodger et al., 2019; Shenker et al., 2015). Hence, in this study, we specifically focused on the four BC molecular subtypes and combined gene methylation with expression profiles to find the common characteristics among different BC types. Arechederra et al. previously reported that hypermethylation could indicate elevated oncogene levels (Arechederra et al., 2018). Therefore, we expected that the screening of hypermethylated and up-regulated oncogenes could identify some potential prognostic biomarkers.

Our study mainly focused on an oncogene: Wilms’ tumor 1 (*WT1*) and the potential mechanism that affecting its expression in the four types of BC. *WT1* was reported to be expressed in 87% of primary BC (Loeb et al., 2001) and was associated with a poor prognosis of BC (Miyoshi et al., 2002). In BC tissues, intragenic regions of *WT1* were hypermethylated (Lian et al., 2012). However, these previous studies about *WT1* were investigated in a single subtype of BC and had no subsequent analysis on its molecular mechanism. Hence, in our study, methylation level and its association with *WT1* expression in different types of BC were analyzed and the underlying oncogenic mechanism of *WT1* was further investigated.

In this study, we downloaded DNA methylation datasets and RNA expression profiles of primary BC tissues from The Cancer Genome Atlas (TCGA) to identify common hypermethylated and up-regulated genes among the four molecular subtypes (luminal A, luminal B, basal-like, and HER2-enriched). Based on the TCGA dataset, we also counted the number of methylated sites and examined the correlation between methylation sites and gene expression. The downstream genes of transcription factors were predicted using the Gene Transcription Regulation Database (GTRD). The prognostic value of signature genes was evaluated using the TCGA dataset and validated by the Gene Expression Omnibus (GEO) dataset. Finally, the protein-protein interaction network was constructed using Search Tool for Interacting Chemicals (STITCH).

## Materials & Methods

### Data collection

We obtained the DNA methylation data of 306 invasive breast carcinoma (BC) samples (beta values from methylation 450 K) from UCSC Xena (https://xenabrowser.net/datapages/?hub=https://tcga.xenahubs.net:443). The invasive BC datasets consisted of 98 normal tissue samples, 108 luminal A primary tumor tissue samples, 46 luminal B primary tumor tissue samples, 40 basal-like primary tumor tissue samples, and 14 HER2-enriched primary tumor tissue samples. The 450 K microarray contains not only CpG and CNG sites, CpG islands/shores/shelves/open sea, non-coding RNA (microRNAs and long non-coding RNAs), and sites surrounding the transcription start sites (−200 bp to −1,500 bp, 5′-UTRs and, exons 1) for coding genes, but also the corresponding gene bodies and 3′-UTR (Sandoval et al., 2011). Based on the platform, the methylation probes can map to a wider variety of gene regions to obtain more data. The gene body region consisted of exons and introns (Han et al., 2017). The island (or CpG island) was defined as a 200-bp stretch of DNA with a C+G content of 50% and an observed CpG/expected CpG of over 0.6 according to that proposed by Gardiner-Garden and Frommer in 1987 (Gardiner-Garden & Frommer, 1987). The sequences up to 2 kb distant CpG islands were termed “CpG island shores” (Irizarry et al., 2009). The sequences from 2 to 4 kb distant CpG islands were denoted as shelves. The rest of the genome was defined as “open sea” (Visone et al., 2019).

We also collected the gene expression profiles from 648 human tissues (including 114 normal tissues, 236 luminal A primary tumor tissues, 132 luminal B primary tumor tissues, 103 basal-like primary tumor tissues, and 63 HER2-enriched tumor tissues), which were sequenced using the Illumina HiSeq 2000 RNA Sequencing platform, from UCSC Xena. The validation cohort (GSE20685), which included 327 primary breast cancer samples, was downloaded from the GEO (https://www.ncbi.nlm.nih.gov/geo/).

### Selecting highly expressed and hypermethylated genes

We identified hypermethylated BC genes using the R package ChAMP. First, we loaded the beta value matrix and patient clinical information (including patient ID and tumor subtype). The champ.filter function was carried out to remove low-quality samples and probes. Second, we determined the NA value in the matrix using the Combine method of the champ.impute function, with a k value of 5, a probe cutoff of 0.1, and a sample cutoff of 0.5. Third, we performed quality control using the champ. QC function to ensure that the loaded data would be available for the subsequent analysis. Type II probe normalization was performed using the BMIQ method of the champ.norm function. Finally, the differentially methylated probes across the four BC subtypes were recognized using the champ.DMP function. They were selected with the following criteria: an absolute value of deltaBeta value > 0.2 and an adjusted *P* value < 0.05 (Chang et al., 2019).

The RNA expression profile of the dataset described above showed a log2 (x+1) transformed RSEM (RNA-Seq expression estimation by Expectation-Maximization) normalized count, which was directly downloaded from Xena (https://xenabrowser.net/datapages/?dataset=TCGA.BRCA.sampleMap%2FHiSeqV2&host=https%3A%2F%2Ftcga.xenahubs.net&removeHub=https%3A%2F%2Fxena.treehouse.gi.ucsc.edu%3A443). Before analysis, we performed gene filtering. Genes with extremely low expression (0 in more than 50% of samples) were removed from the subsequent analysis. We calculated the mean value for each gene in normal and tumor tissue, as well as the fold change of log2 (mean value of tumor tissue/mean value of normal tissue). The *P* value was computed using the t. test function and adjusted by FDR (false discovery rate) with p.adjust function in R. The gene with |log2 (fold change) |>1 and adjusted *P* value <  0.05 were defined as dysregulated genes.

### Correlation and survival analysis

We calculated the correlation coefficient for methylation sites and gene expression and analyzed the gene co-expression using the Spearman’s rank correlation test.

Survival analysis was performed in R Studio with the survminer package via the Cox proportional hazards regression model and visualized by survival package. After inputting patients’ survival time and the endpoint information (dead or alive), Kaplan–Meier analysis was used to obtain the survival curve. *P* values were calculated using the log-rank *t*-test.

### The download of breast cancer-related genes and human oncogenes

The gene list, containing 228 genes that have been reported to affect the development of BC, was downloaded from the Disease gene search engine (DigSee, http://210.107.182.61/geneSearch/). DigSee is a web tool developed to search MEDLINE abstracts for evidence sentences depicting that genes take part in the development of cancers via biological events (Kim et al., 2013). After selecting a type of cancer on DigSee, it will return all genes related to the cancer type and the corresponding references of each gene. On DigSee, 7,449 genes are documented for BC. We chose 228 genes that were supported by at least 10 references in BC to screen for the target genes of this study. The BC-related genes queried on DigSee were listed in Table S1.

The human oncogenes were downloaded from the Oncogene database (http://ongene.bioinfo-minzhao.org/download.html). Oncogene database is a literature-based genetic resource ground on a comprehensive review of research literature about oncogenes (Liu, Sun & Zhao, 2017). A total of 802 human oncogenes are recorded on the Oncogene database (Table S2). They were used to further screen for target genes of this study.

### *WT1* potential target genes prediction

We predicted the *WT1* target genes using GTRD (http://gtrd.biouml.org/). GTRD is a database that began in 2011, which contains transcription factor binding sites identified by ChIP-seq experiments for *Homo sapiens* (Yevshin et al., 2019). Using GTRD, we set transcription factor binding site location at promoter [−1000, +100] to predict its target genes. The gene list was shown in Table S3.

### Protein-protein interaction network construction

The protein-protein interaction network was generated using STITCH (http://stitch.embl.de/). STITCH is a web to explore the intersections between proteins and small molecules. It integrates these disparate data sources for 430,000 chemicals into a single, easy-to-use resource (Szklarczyk et al., 2016). After entering a gene, it will return its related genes and drugs.

### Immune infiltration analysis

We analyzed the immune infiltration fraction according to the method as mentioned by Thorsson et al. (2018), which estimated the relative fraction of 22 immune cells of each patient via CIBERSORT (Newman et al., 2015). CIBERSORT is a calculation method that can accurately calculate the relative level of multifarious immune cell types in a mixture of compound gene expression. CIBERSORT uses the gene expression signatures of 547 genes (LM22 files) as the input matrix to characterize and quantify immune cell subtypes. Using the TCGA’s primary BC tissues RNA expression data and LM22 files as input data, CIBERSORT was implemented in “relative mode” to estimate the relative abundance of tumor-infiltrating immune cells. Next, we calculated the difference in immune infiltration between various groups using an unpaired *t*-test.

### Statistical analysis

All statistical analyses in our study were carried out using R. During the selection of highly expressed genes, the *P* value was computed using the t. test function and adjusted by FDR (false discovery rate) with p.adjust function in R. In survival analysis, *P* values were calculated using the log-rank *t*-test. The difference in immune infiltration between various groups was calculated using an unpaired *t*-test.

## Results

### Selecting highly expressed, hypermethylated genes across the four BC subtypes

**Figure 1:**
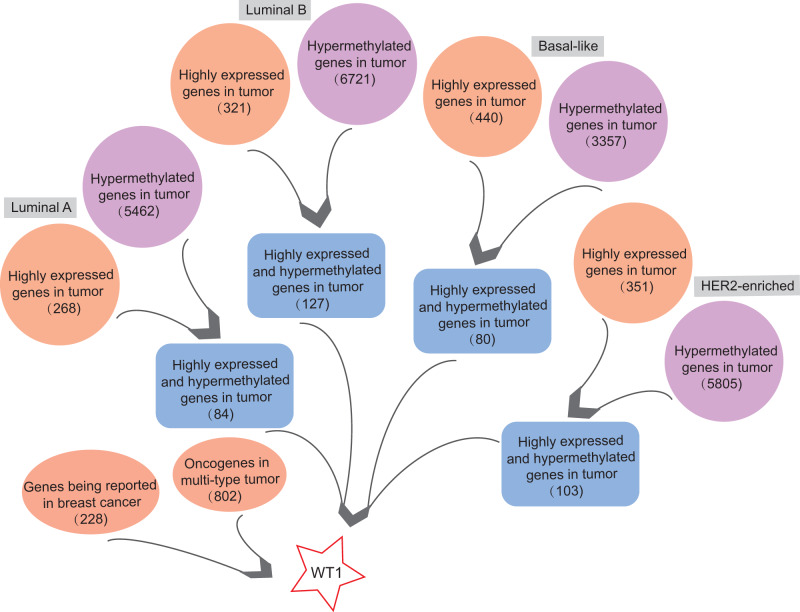
The workflow. Orange bubbles presented the number of highly expressed genes in different subtypes of BC based on TCGA RNA-seq analysis. Purple bubbles presented the number of hypermethylated genes in different subtypes of BC based on TCGA methylation data. Blue boxes showed the number of both up-regulated and hypermethylated genes in four subtypes of BC based on TCGA data. The left red bubble presented the number of gene reported in BC, while the right red bubble showed oncogene number in multi-type tumors. Tumor sample size of luminal A, luminal B, Basal-like, and HER2-enriched BC subtype is 108, 46, 40, and 14, respectively. Normal sample size is 98.

In order to find highly expressed and hypermethylated genes across the four BC subtypes, the differential analysis of gene expression and methylation levels were performed in R studio based on the TCGA’s BC primary tissue RNA-seq and methylation datasets. We found 268, 321, 440, and 351 highly expressed genes and 5,462, 6,721, 3,357, and 5,805 hypermethylated genes in luminal A, luminal B, basal-like, and HER2-enriched BC tumor tissues, respectively (Fig. 1). Next, we selected genes that were both highly expressed and hypermethylated in BC tumor tissues. We found 84, 127, 80, and 103 genes that were highly expressed and hypermethylated in luminal A BC, luminal B BC, basal-like BC, and HER2-enriched BC, respectively (Fig. 1, Table S4). We downloaded 228 BC-related genes from DigSee and 802 oncogenes from the Oncogenes database. In order to find oncogenes that were highly expressed and hypermethylation across the four types of BC, we selected the genes commonly present in all the six gene lists (228 BC-related genes, 802 oncogenes, and highly expressed and hypermethylated genes in the four subtypes of BC). Ultimately, there was only one remaining gene which was the transcription factor Wilms tumor gene 1 (*WT1*). Therefore, *WT1* was chosen for the subsequent analysis.

### *WT1* methylation statuses at various methylation sites

**Figure 2:**
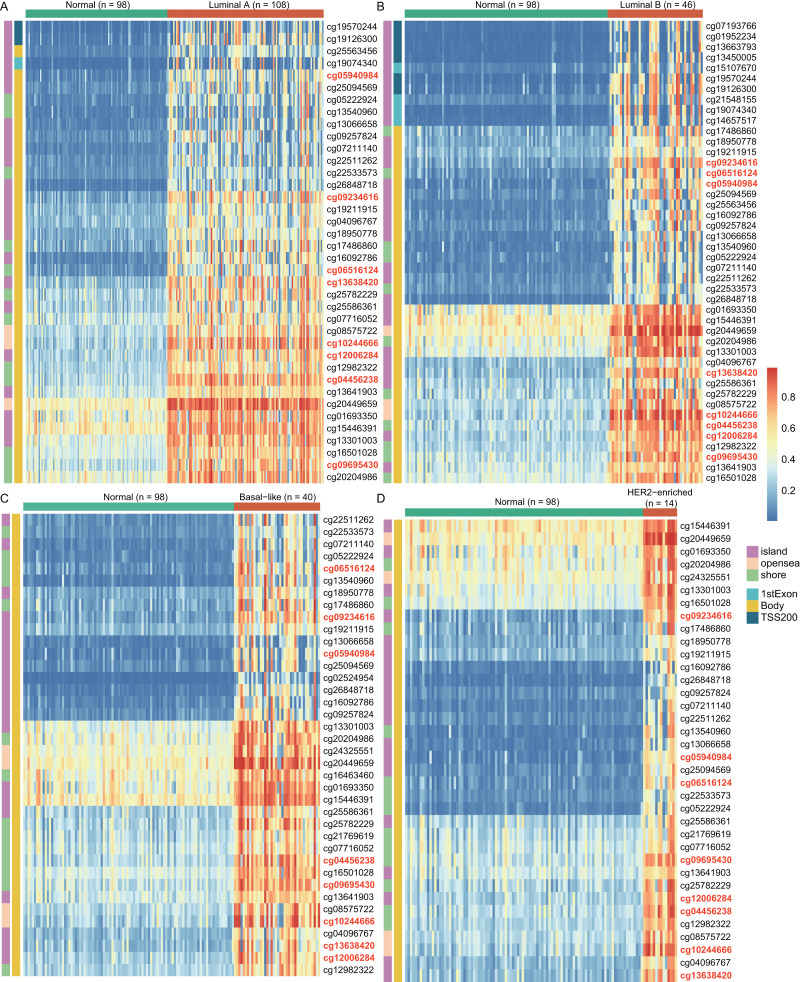
The differentially *WT1* methylated sites in the four BC subtypes from TCGA methylation data (A-D). In the heatmap, each column presents one patient and each row is a cg probe. The left bars show cg probe’s genomic region. The deeper red means higher methylation levels.

To explore the cause of high *WT1* expression, we counted the methylated sites located on *WT1* based on the TCGA’s BC primary tissue methylation datasets. For the four BC subtypes (luminal A, luminal B, basal-like, and HER2-enriched), the number of *WT1*-methylated sites were 38, 44, 38, and 36, respectively. Figure 2 showed the methylation levels of statistically discrepant methylated sites in luminal A (Fig. 2A), luminal B (Fig. 2B), basal-like (Fig. 2C), and HER2-enriched BCs (Fig. 2D). In the heatmap, each column represents an individual patient and each row is a cg probe. The left bar shows the cg probe’s genomic region. The deep red color stands for high methylation level. We found that all methylation sites showed high methylation levels, particularly cg09695430, cg05940984, cg06516124, cg12006284, cg13638420, cg09234616, cg04456238, and cg10244666. Most of the methylated sites in luminal A, luminal B, basal-like, and HER2-enriched BCs were located in the body regions, and the percentages were 92.1% (35/38), 77.3% (34/44), 100% (38/38), and 100% (36/36), respectively. These might explain the strong *WT1* expression levels in BC tissues (Yang et al., 2014).

**Figure 3:**
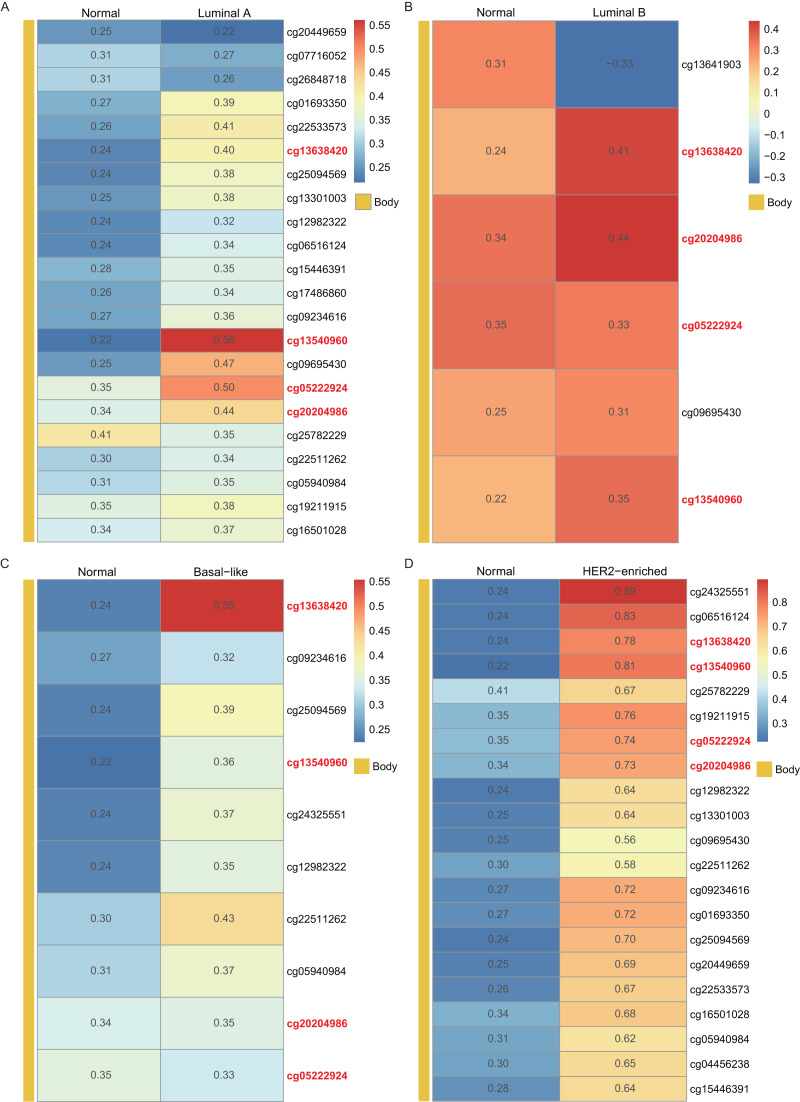
The correlation of *WT1* methylated sites and *WT1* expression in four subtypes of BC was observed based on TCGA data (A-D). The number in each small box was the correlation coefficient of *WT1* expression and the methylated sites. The deeper red means higher correlation. The bar in the left showed the genomic region of each cg probe.

Using the *WT1* RNA expression profiles of the four types of BC primary tumor tissue downloaded from TCGA, we analyzed the correlation between *WT1* expression and the methylated sites. In Fig. 3, the number displayed in each cell is the correlation coefficient of *WT1* expression and the methylated sites. The deep red color stands for a strong correlation. The bar on the left shows the genomic region of each cg probe. Cg13540960, cg05222924, cg20204986, and cg13638420 had fair positive associations (Akoglu, 2018) (correlation coefficient >  0.3, *P* <  0.05) with *WT1* expression in all the BC subtypes (Fig. 3). HER2-enriched BC had the highest correlation coefficient (median = 0.69) of all the common methylated sites. In contrast, luminal A, luminal B, and basal-like subtypes had median correlation coefficients of 0.36, 0.34, and 0.36, respectively.

**Figure 4:**
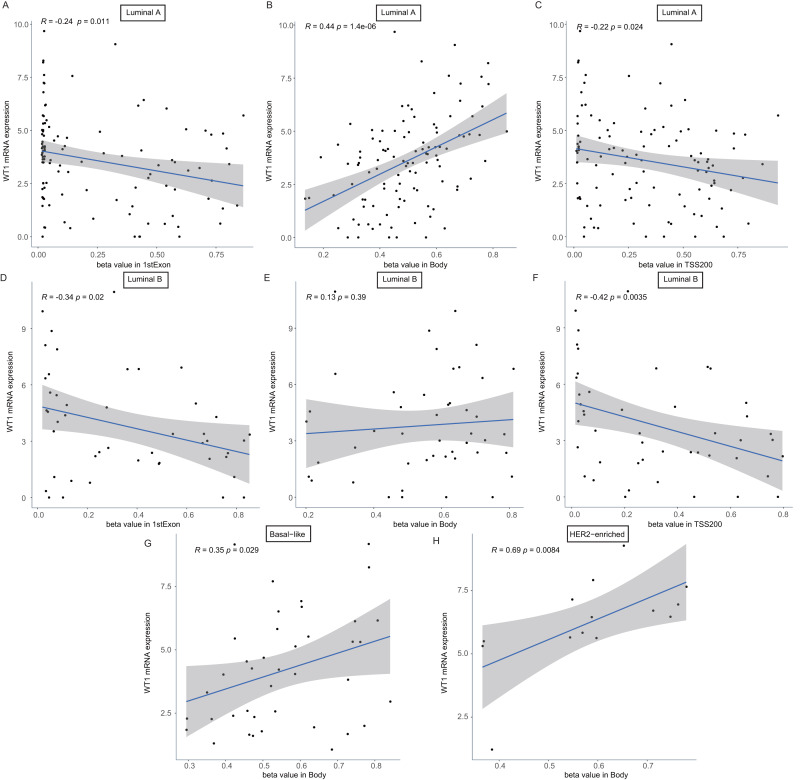
The association analysis between beta value of 1stExon, Body or TSS200 region and *WT1* expression in luminal A (A–C), luminal B (D–F), basal-like (G) or HER2-riched (H) BC based on TCGA data.

To confirm whether the methylation levels of the different genomic regions were related to *WT1* expression, we calculated the mean beta value (an indicator of methylation level) of each patient in the 1stExon, Body, and TSS200 regions and performed correlation analysis between beta value and *WT1* expression based on the TCGA’s BC primary tissue RNA-seq and methylation datasets. In luminal A and luminal B BCs, *WT1* expression was negatively associated with the methylation level of the 1stExon (Figs. 4C and 4D) and TSS200 regions (Figs. 4C and 4F). In luminal A, basal-like, and HER2-enriched BCs, *WT1* expression was positively associated with the methylation level of the gene body (Figs. 4B, 4G and 4H). However, in luminal B BC, *WT1* expression showed no statistically significant association with the methylation level of the gene body (Fig. 4E).

### Correlation analyses of the expression of *WT1* and its downstream genes

**Figure 5:**
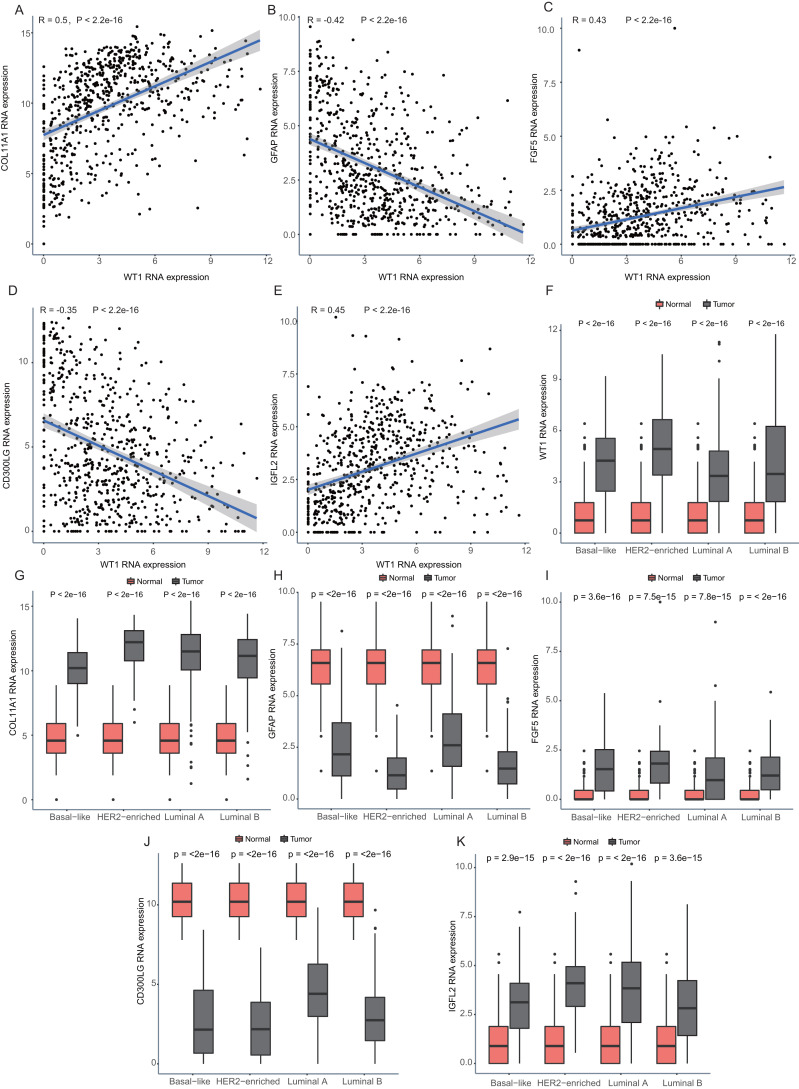
Correlation analyses and gene expression in the four BC subtypes based on TCGA RNA-seq profiles. (A–D) Correlation analysis showed *WT1* expression had a correlation with the downstream genes *COL11A1*, *GFAP*, *FGF5*, * CD300LG*, and *IGFL2*. (E–I) *WT1*, *COL11A1*, *GFAP*, *FGF5*, *CD300LG*, and *TDO2* expression was shown across the four BC subtypes of BC and normal tissues.

**Figure 6:**
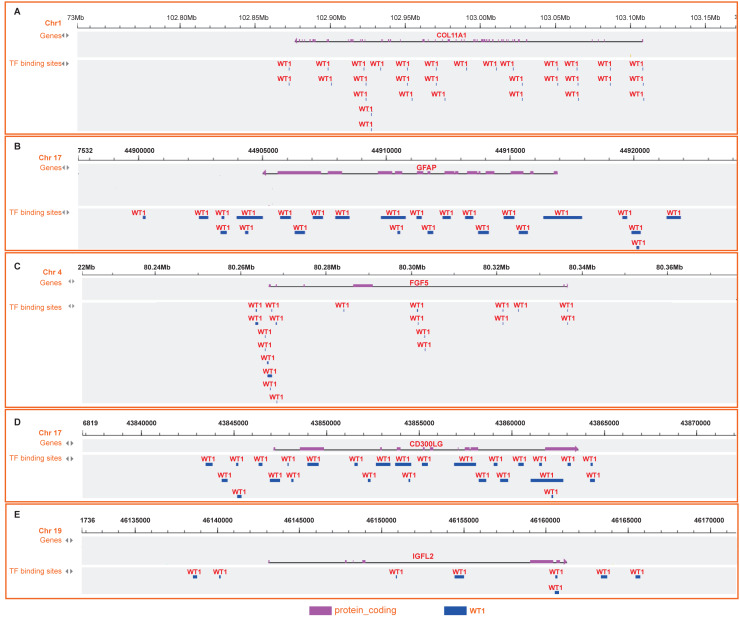
(A-E) *WT1* binding sites with its potential downstream genes predicted on GTRD.

Since *WT1* is a transcription factor, we further examined the genes regulated by *WT1*. A total of 17,214 genes were predicted as *WT1-* target genes (set transcription factor binding site location on promoter [−1000,  + 100]) using GTRD. We then calculated the coefficient of association between the expression of the differentially expressed genes and *WT1* in the four types of BC tumors to identify *WT1*’s co-expressed genes based on the TCGA’s BC primary tissue RNA-seq dataset. After screening with the selection criteria (*P* value < 0.05 and —correlation coefficient—>0.35), we identified 77, 223, 239, and 279 genes from luminal A, luminal B, basal-like, and HER2-enriched BC groups, respectively. Among these, we found 5 shared genes, including *COL11A1*, *GFAP*, *FGF5*, *CD300LG*, and *IGFL2*. Figs. 5A–5E showed the correlation of them with *WT1* expression, of which three genes exhibited fair positive correlations and two genes showed negative correlations (including *GFAP*). A comparison of the gene expression levels in BC tissues and normal tissues was shown in Figs. 5F–5K. *WT1*, *COL11A1*, *FGF5*, and *IGFL2* were up-regulated in tumor tissues, while *GFAP* and *CD300LG* were down-regulated. We also calculated the correlation coefficient of *COL11A1*, *GFAP*, and *FGF5* with *WT1* expression in GSE20685. *COL11A1* expression was fairly positively correlated with *WT1* expression and the expression of *FGF5* was poorly positively correlated with *WT1* expression. *GFAP* and *WT1* expressions had no statistically significant correlation (Fig. S1). The *WT1* binding sites of its potential downstream genes were shown in Fig. 6. The binding sites were predicted by GTRD based on ChIP-seq experiment. All the five genes possessed binding sites upstream of their protein-coding regions.

### Survival and protein-protein interaction network analysis

**Figure 7:**
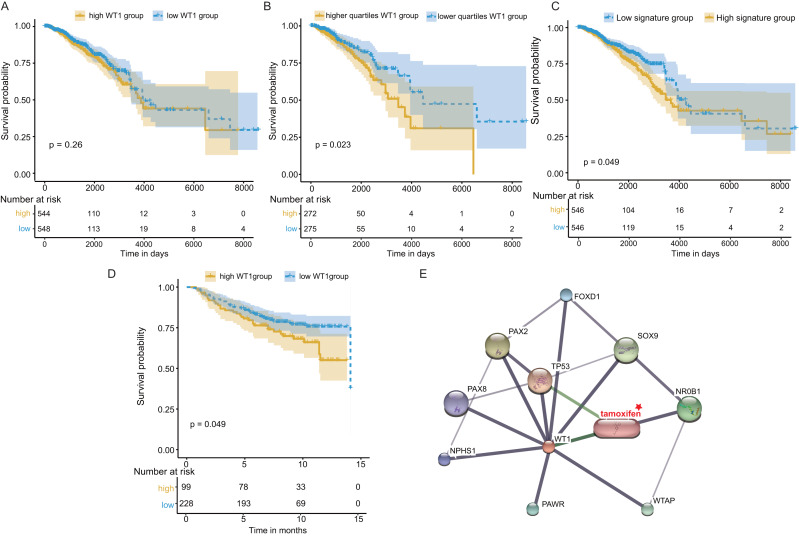
Survival and protein-protein interaction network analysis. (A) Survival analysis between the high and low *WT1* expression group was performed using TCGA RNA-seq and survival data. (B) Survival analysis between the upper and lower quartile *WT1* expression group was carried out using TCGA RNA-seq and survival data. (C) Survival analysis between the high and low signature expression group was performed with TCGA RNA-seq and survival data. (D) Survival analysis between the high and low *WT1* expression group in the validation cohort (GSE20685). (E) *WT1* protein-protein interaction network visualized on STITCH.

Next, we examined *WT1*’s prognostic value based on the TCGA’s BC primary tissue RNA-seq and survival datasets. When the median *WT1* expression in tumor tissue was set as the cutoff value to divide patients into high- and low-*WT1* expression groups, we observed no statistically significant differences in overall survival (OS) between the two groups using the log-rank test (*P* = 0.26, Fig. 7A) and the Cox regression test (hazard ratio (HR) = 1.2, *P* = 0.26, Table 1). Therefore, we adjusted the cutoff value to the quartiles of *WT1* expression. We found that patients with *WT1* expression below the lower quartile showed better OS rates than patients with *WT1* expression above the upper quartile, and the log-rank test and Cox regression test (HR = 1.68) *P* values were 0.023 and 0.024, respectively (Fig. 7B, Table 1). When we combined the 5 co-expressed genes and *WT1* as a gene signature to predict OS, the patients with low expression levels (favorable 6-gene signature) exhibited better OS compared to patients with high expression levels (unfavorable 6-gene signature). The log-rank test and Cox regression test (HR = 1.38) *P* values were 0.049 and 0.049, respectively (Fig. 7C, Table 1). The validation cohort showed a similar result, with *P* = 0.049 and HR = 1.55 (Fig. 7D, Table 1). Additionally, we used STITCH to construct the protein-protein interaction network and showed that *WT1* was linked with tamoxifen, a drug for BC treatment (Fig. 7E).

### Immune infiltration analysis

**Figure 8:**
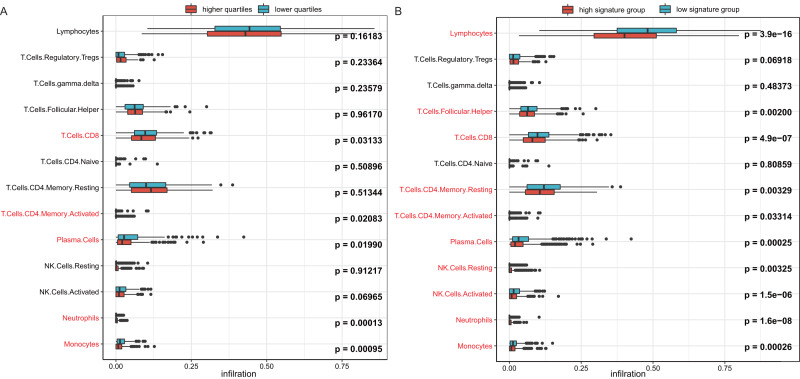
Immune infiltration. (A) Comparison between the immune infiltration of the lower quartile *WT1* expression group and the higher quartile group using TCGA RNA-seq profiles. (B) The immune infiltration of the low 6-gene signature group and the high 6-gene signature group using TCGA RNA-seq profiles.

To investigate the reason behind the better outcomes of the lower quartile *WT1* group and the favorable 6-gene signature group, we evaluated the differences in tumor microenvironments between groups based on the TCGA’s BC primary tissue RNA-seq dataset. The infiltration rates of the CD8 T cells, plasma cells, and monocytes in the lower quartile *WT1* group were significantly higher than that of the higher quartile *WT1* group (Fig. 8A). The CD8 T cells, plasma cells, and monocytes in the favorable 6-gene signature group also had significantly higher infiltration rate than that of the unfavorable 6-gene signature group (Fig. 8B). Moreover, the lymphocytes, follicular helper T cells, and activated NK cells had higher infiltration rates in the favorable 6-gene signature group (Fig. 8B).

**Table 1:** Hazard ratio and *P* value of each group.

**Data source**	**Group**	**Hazard ratio (HR)**	*P***value**	**Lower 95% CI range**	**upper 95% CI range**
TCGA	High WT1 group	1.2	0.26	0.87	1.65
TCGA	High quartiles WT1 group	1.68	0.024	1.07	2.65
TCGA	High signature group	1.38	0.049	1.0	1.90
GSE20685	High WT1 group	1.55	0.049	0.99	2.42

## Discussion

In this study, we analyzed the DNA methylation datasets and RNA expression profiles generated by RNA-seq across four molecular subtypes of BC. We found 84, 127, 80, and 103 genes that were highly expressed and hypermethylated in luminal A, luminal B, basal-like, and HER2-enriched BCs, respectively. *WT1,* a gene that was both highly expressed and hypermethylated across all the four subtypes, was selected as a potential prognostic biomarker of BC. McGregor et al. has previously reported that *WT1* expression was up-regulated in BC cells (McGregor et al., 2018), which was consistent with our findings. Furthermore, we found that *WT1* exhibited hypermethylation in BC tissues, which was consistent with the results reported by Kim et al. (2012). Compared to Kim’s research which was performed on BC cells and a single subtype of BC, our study additionally showed that *WT1* was highly expressed and hypermethylation in all the four subtypes of BC. Besides, this is the first research reporting the association of *WT1* expression and methylation in the four subtypes of BC. Since DNA methylation in the promoter region can suppress gene expression but up-regulate gene expression in the gene body region (Yang et al., 2014), we further examined the detailed distribution of the methylated sites. As expected, the *WT1* methylation sites were mostly or all located in the gene body region. In luminal A, basal-like, and HER2-enriched BCs, *WT1* expression was positively associated with the methylation level of the gene body, supporting that gene body methylation is related to high expression of *WT1*. This phenomenon might be owing to the modulation effects of gene body methylation on the binding of transcription factor to the promoter region (Wang et al., 2016). However, in luminal B BC, *WT1* expression showed no statistically significant association with the methylation level of the gene body, probably due to the fair negative correlation of cg13641903 methylation with *WT1* expression. A possible reason to explain the up-regulation of *WT1* in luminal B BC is that L1 transposon subfamilies that are up-regulated in ER+/HER- BC (Yandim & Karakulah, 2019) could regulate *WT1* gene expression (Ramos et al., 2011). Given that luminal B BC was characterized by ER+ and/or PR + as well as HER2-, hence, we supposed that *WT1* expression was influenced more by repetitive DNA in luminal B BC. This inspires scientists to put attention not only on the dysregulation at the gene level but also on the repetitive DNA.

To further understand the *WT1* regulatory mechanism, we predicted its downstream genes using GTRD. We confirmed that 5 genes, including *COL11A1*, *GFAP*, *FGF5*, *CD300LG*, and *IGFL2*, were co-expressed with *WT1*. In agreement with our results, the high expressions of *WT1*, *COL11A1*, and *FGF5* in BC tumors have also been reported in previous literatures (Huang, Wang & Yang, 2018; Li, Kong & Zou, 2017a; Malvia et al., 2019). *WT1* overexpression could promote ERK1/2 phosphorylation and thus decreasing E-cadherin expression and enhance EMT (epithelial-to-mesenchymal transition) (Han et al., 2020). Its potential downstream gene *COL11A1* was also EMT-related: *COL11A1* and *CK7* co-expression indicated that the cell is undergoing EMT process (Garcia-Pravia et al., 2013); *COL11A1* deficiency significantly induced the expression of E-cadherin (one of the epithelial markers) (Zhang et al., 2018). The induction of *GFAP* might be caused by the reduction of pERK (Lind et al., 2006). Therefore, we supposed that *WT1* might affect EMT by regulating *COL11A1* and *GFAP* expressions. *CD300LG* has diverse immunological functions and is capable of recognizing and interacting with extracellular lipids (Borrego, 2013; Stoy et al., 2015). *WT1* induces the generation of WT1-specific CD8+ T cells (Lu et al., 2018), suggesting that *WT1* and *CD300LG* have a functional dependency. *IGFL2* is a member of the insulin-like growth factor family, which plays a key role in cell energy metabolism, growth, and development, especially in prenatal growth (Emtage et al., 2006). *IGFL2* was up-regulated in the four types of BC in our result, which might contribute to tumor cell growth. Besides, *COL11A1* expression has been suggested as a promising marker for invasive breast lesions (Freire et al., 2014) and ovarian cancer and has a positive correlation with cisplatin treatment (Rada et al., 2018).

For *WT1*’s prognostic value, our results showed that the lower quartile *WT1* group exhibited better OS compared to the upper quartile *WT1* group based on the TCGA dataset, which was in agreement with a previous study (Artibani et al., 2017). Artibani et al. concluded that the poor prognosis for patients with high *WT1* expression may be caused by EMT. The expression of *COL11A1*, a downstream gene of *WT1*, has shown a correlation with the invasive capacity of BC cells. This may be one of the reasons for the poor prognosis of the upper quartile *WT1* group and the unfavorable 6-gene signature group. Additionally, *WT1* as a tumor-associated antigen stimulates the growth of WT1-specific CD8^+^ cytotoxic T cells (Lehe et al., 2008) which is associated with better BC outcomes (Ziai et al., 2018). However, the presence of regulatory T cells could inhibit the induction of anti-WT1-126 CD8+ CTL responses (Lehe et al., 2008). The depletion of CD4+CD25+ regulatory T cells is necessary for the generation of an effective WT1-specific cytotoxic response (Asemissen et al., 2006). In our immune infiltration analysis (Fig. 8), regulatory T cells were observed both in the upper/lower quartile *WT1* groups and the favorable/unfavorable signature groups, which might result in the inhibition of WT1-specific CD8+ T cell generation. CD8+ tumor-infiltrating lymphocytes demonstrated prognostic benefit only when present in combination with plasma cells (Kroeger, Milne & Nelson, 2016). The relatively higher plasma cell infiltrations in the lower quartile *WT1* group and the favorable signature group may, therefore, be a factor leading to the better outcomes of these groups. Finally, the protein-protein interaction network constructed using STITCH demonstrated an association between *WT1* and tamoxifen. Han et al. (2008) also found that *WT1* could regulate the resistance to antiestrogen, including tamoxifen.

## Conclusion

In conclusion, we showed that *WT1* is highly expressed and hypermethylated in the four BC subtypes. All the *WT1* methylation sites exhibited hypermethylation and most of them were located in the gene body region. Methylation levels of the TSS200 and 1stExon regions were negatively correlated with *WT1* expression in luminal A and luminal B BCs, while that of the gene body was positively associated with *WT1* expression in luminal A, basal-like, and HER2-enriched BCs. Furthermore, we found five dysregulated genes, including *COL11A1*, *GFAP*, *FGF5*, *CD300LG*, and *IGFL2*, in the four BC types that was predicted as *WT1*’s downstream genes. When we used the expression of the five genes and *WT1* as a signature, the group with low expression exhibited better OS than the high expression group.

##  Supplemental Information

10.7717/peerj.11377/supp-1 Supplemental Information 1 The calculation of the correlation coefficient of COL11A1, GFAP, and FGF5 with WT1 expression in GSE20685

10.7717/peerj.11377/supp-2 Supplemental Information 2 The genes that had been reported in breast cancer

10.7717/peerj.11377/supp-3 Supplemental Information 3 The list of Oncogenes

10.7717/peerj.11377/supp-4 Supplemental Information 4 WT1 potential downstream gene list

10.7717/peerj.11377/supp-5 Supplemental Information 5 The highly expressed hypermethylated genes

## Additional Information and Declarations

### Data Availability

The following information was supplied regarding data availability: Data is available at NCBI GEO: GSE20685 and at Xena. Link to Xena: https://xenabrowser.net/datapages/?dataset=TCGA.BRCA.sampleMap%2FHiSeqV2host=https%3A%2F%2Ftcga.xenahubs.netremoveHub=https%3A%2F%2Fxena.treehouse.gi.ucsc.edu%3A443.
