# Supplementary material for: Comprehensive analysis based on DNA methylation and RNA-seq reveals hypermethylation of the up-regulated WT1 gene with potential mechanisms in PAM50 subtypes of breast cancer
Source: PeerJ. 2021 May 4;9:e11377. doi: 10.7717/peerj.11377 (PMC8103922; doi:10.7717/peerj.11377)
Supplement: Supplemental Information 1 [file peerj-09-11377-s001.docx]

**Supplementary Figure S1. The calculation of the correlation coefficient of COL11A1, GFAP, and FGF5 with WT1 expression in GSE20685.**

**
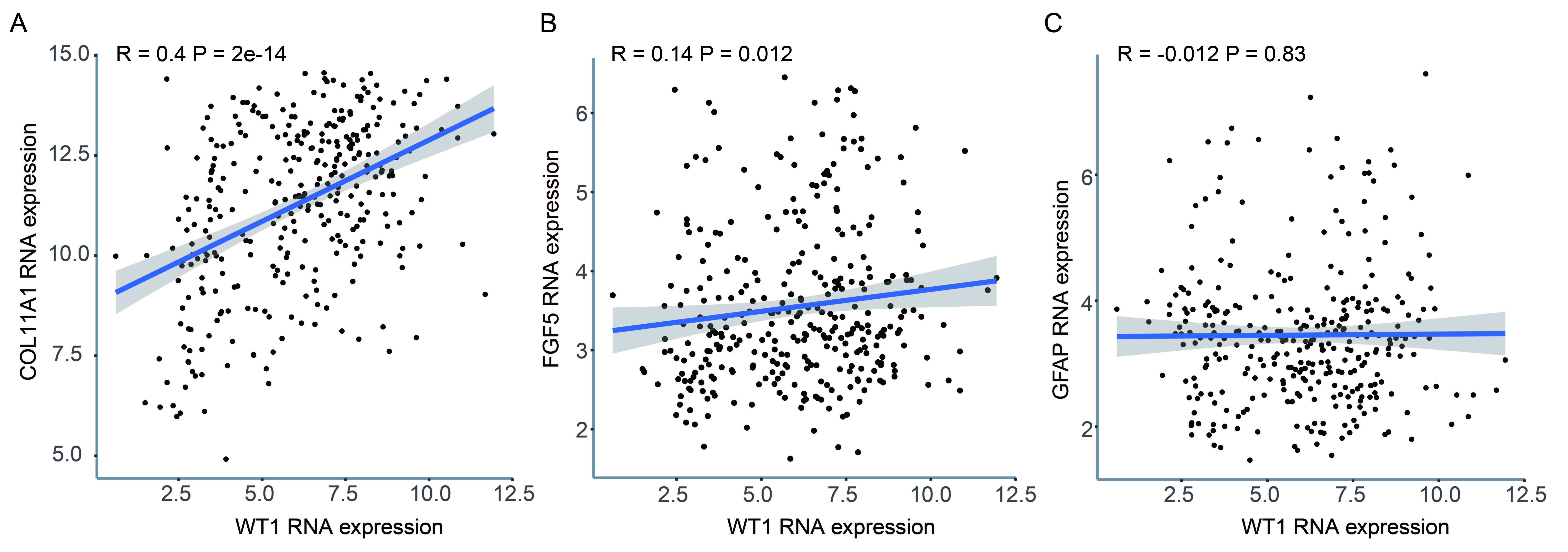
**

**Supplementary Figure S1. The calculation of the correlation coefficient of COL11A1, GFAP, and FGF5 with WT1 expression in GSE20685.**
